# When chronic obstructive pulmonary disease meets small cell lung cancer: an unusual case report of rapid progression

**DOI:** 10.1186/s12877-023-04508-7

**Published:** 2023-12-11

**Authors:** Xu Zhang, Jia Zeng, Xiyu Huang, Zhishu Li

**Affiliations:** 1 Department of Respiratory and Critical Care Medicine, Guangyuan Central Hospital, 10 Lianhua Road, Lizhou District, Guangyuan City, 628000 Sichuan Province China; 2 Sichuan Academy of Medical Sciences, Cardiac Surgery Center, Sichuan Provincial People’s Hospital, Chengdu, 610000 China; 3 Department of Respiratory and Critical Care Medicine, Tianjin Medical University General Hospital, Tianjin, 300000 China

**Keywords:** COPD, SCLC, progression, Survival outcome, Case report

## Abstract

**Background:**

Chronic obstructive pulmonary disease (COPD) is a chronic inflammatory disease and a risk factor for lung cancer. Small cell lung cancer is a neuroendocrine tumor with a high degree of malignancy and an overall five-year survival rate of less than 7%.

**Cases presentation:**

Herein, we report the case of an 68-year-old male presented to the respiratory department with cough, sputum, and dyspnea.

He was diagnosed as community acquired pneumonia and treated with intravenous anti-infection. Previous pulmonary function was definitively diagnosed as COPD. About 7 months after discharge, the patient returned to the hospital for cough and dyspnea. After diagnosis of the tumor, cisplatin, etoposide and durvalumab were administered. Finally the patient died of respiratory failure approximately 9 months after his diagnosis.

**Conclusions:**

For COPD patients with immunocompromised manifestations, it is necessary to be alert to complications and shorten the follow-up interval of chest CT. COPD may accelerate the formation and progression of SCLC.

## Introduction

Lung cancer is a common complication and the main cause of death of chronic obstructive pulmonary disease (COPD). Common causes of the two diseases include not only tobacco exposure, but also epigenetic changes in DNA methylation, genetic susceptibility and other mechanisms that are considered to be important potential factors in the development of lung cancer [1]. In particular, the prognosis of patients with extensive small cell lung cancer (ES-SCLC) is extremely serious. ES-SCLC has a high degree of malignancy, easy recurrence and short survival. The long-term survival of ES-SCLC is very difficult. In recent years, immunotherapy has shown certain effects in improving efficiency, efficacy and prolongation of survival, but it is still unsatisfactory [2]. In conclusion, from mechanism to treatment, COPD combined with SCLC urgently needs to be paid attention to and more effective treatment methods are sought.

## Case presentation

**Fig. 1:**
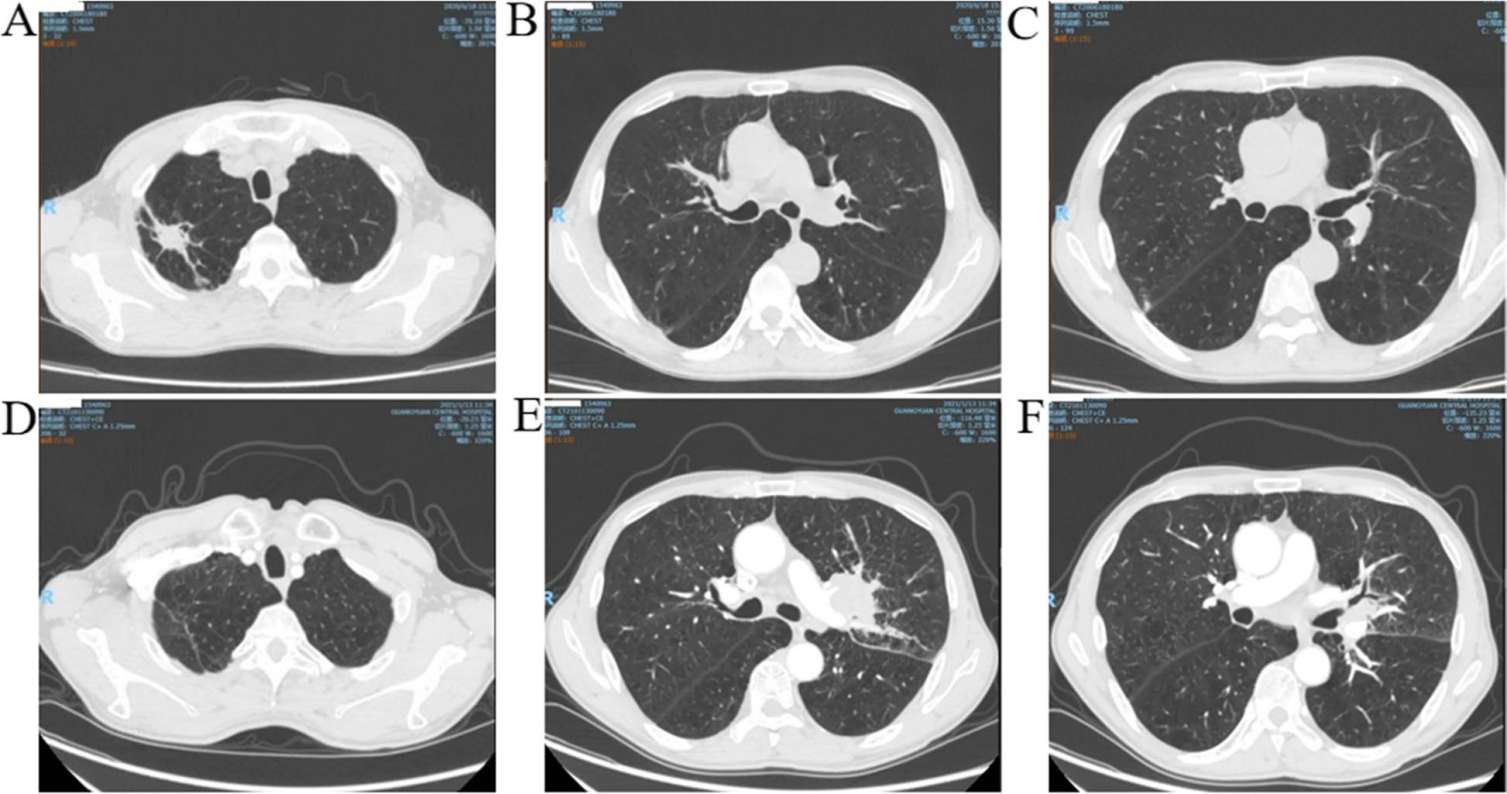
**A**, **B**, and **C** are the chest CT scan results from the first admission. **D**, **E**, and **F** show the results of chest CT scan at the same layer from the second hospitalization

This is a case of a 68-year-old male smoker (50 pack-year history). The occupation of the patient is a farmer. No family history of cancers or lung cancer. He was admitted to hospital due to cough, sputum and dyspnea. He had a 4-year history of COPD and a 3-year history of well-controlled chronic gastritis. He had no other medical conditions such as high blood pressure, diabetes, obesity or autoimmune disease and denied drinking alcohol. Physical examination revealed a barrel-chest.Wet rales and wheezing could be heard in the right upper lung. Laboratory data showed WBC 11.90/10^9^/L (reference, 4–10/10^9^/L), Neu 8.75/10^9^/L (reference, 2.5–7.5/10^9^/L), Lym 0.94/10^9^/L (reference, 1.5–3.3/10^9^/L), CRP 149.77 mg/L (reference, ≤ 10 mg/L). Blood gas analysis PO2 77.10 mmHg (reference, 80-100 mmHg). Chest computerized tomographic (CT) scan revealed signs of emphysema, irregular mass-like consolidation with cavity shadows in the right upper lobe, and thickening of local interlobular septa (Fig. 1A). Bronchoscopy and alveolar lavage showed no abnormality. Combined with clinical manifestations, laboratory examination and imaging data, he was diagnosed as community acquired pneumonia (CAP). He was treated with intravenous ceftizoxime (1 g/Q12h/9d) for anti-infection, intravenous methylprednisolone (40 mg/qd/5d), atomized salbutamol for antispasmosis, relief of bronchoscpasm, cough and expectorant treatment, and was discharged with improvement. Pulmonary function testing showed a decreased FEV1/FVC ratio (53.47), an FEV1 of 1.08 L (45% normal), and reduced diffusing capacity of 23%. After discharge, he was treated with LABA/LAMA and recovered well. He did not return to the hospital for follow-up.

About 7 months after discharge, the patient returned to the hospital for cough and dyspnea. Physical examination of the left upper lung breathing sound was low. Laboratory data showed a cytokeratin 19 fragment of 2.42 ng/ml (reference, < 3.3 ng/ml) and neuron-specific enolase 85.87 ng/ml (reference, 0–16.3 ng/ml). Contrast-enhanced chest CT showed resolution of the right upper lung lesion and residual scarring (Fig. 1D). Irregular mass in the bronchial opening area of the upper lobe of the left lung and fusion with enlarged lymph nodes in the left hilum and adjacent mediastinum: Central lung cancer of the left lung with left pulmonary obstructive inflammation, lymph node metastasis in the mediastinum and left hilum, and arteriovenous invasion of the left upper lung were considered to be highly possible (Fig. 1E and F). At the same time, we compared the images of the first hospitalization at the same layers (Fig. 1B and C).

**Fig. 2:**
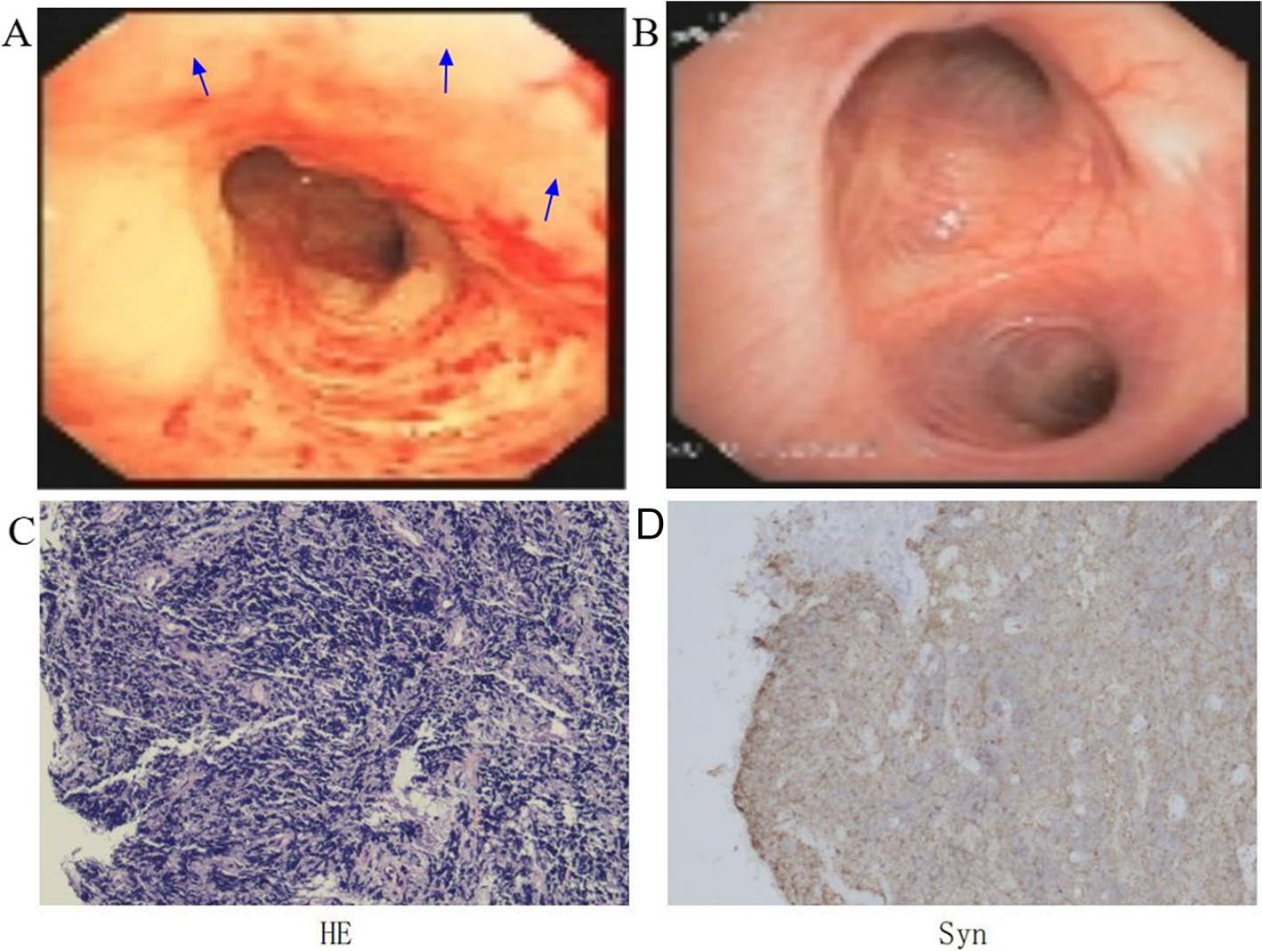
**A** and **B** show the opening of the left upper lobe bronchus at the second and first hospitalization, respectively. **A** also shows the lesion on the bronchoscopic image (blue arrow). **C** shows Hematoxylin–eosin staining, **D** shows immunohistochemistry with Synaptophisin Antibody

Positron emission tomography/CT did not detect an increase in standard uptake value except the lung. Fibrobronchoscopy revealed infiltrating growth of new organisms in the bronchial mucosa of the left upper lobe of the lung covered with white membrane-like neoplasm (Fig. 2A). We compared images of the same site at the first admission (Fig. 2B). Biopsy diagnosed SCLC (Fig. 2C and D). This patient PD-L1 Tumor Proportion Score was 52%. The diagnosis was ES-SCLC.

**Fig. 3:**
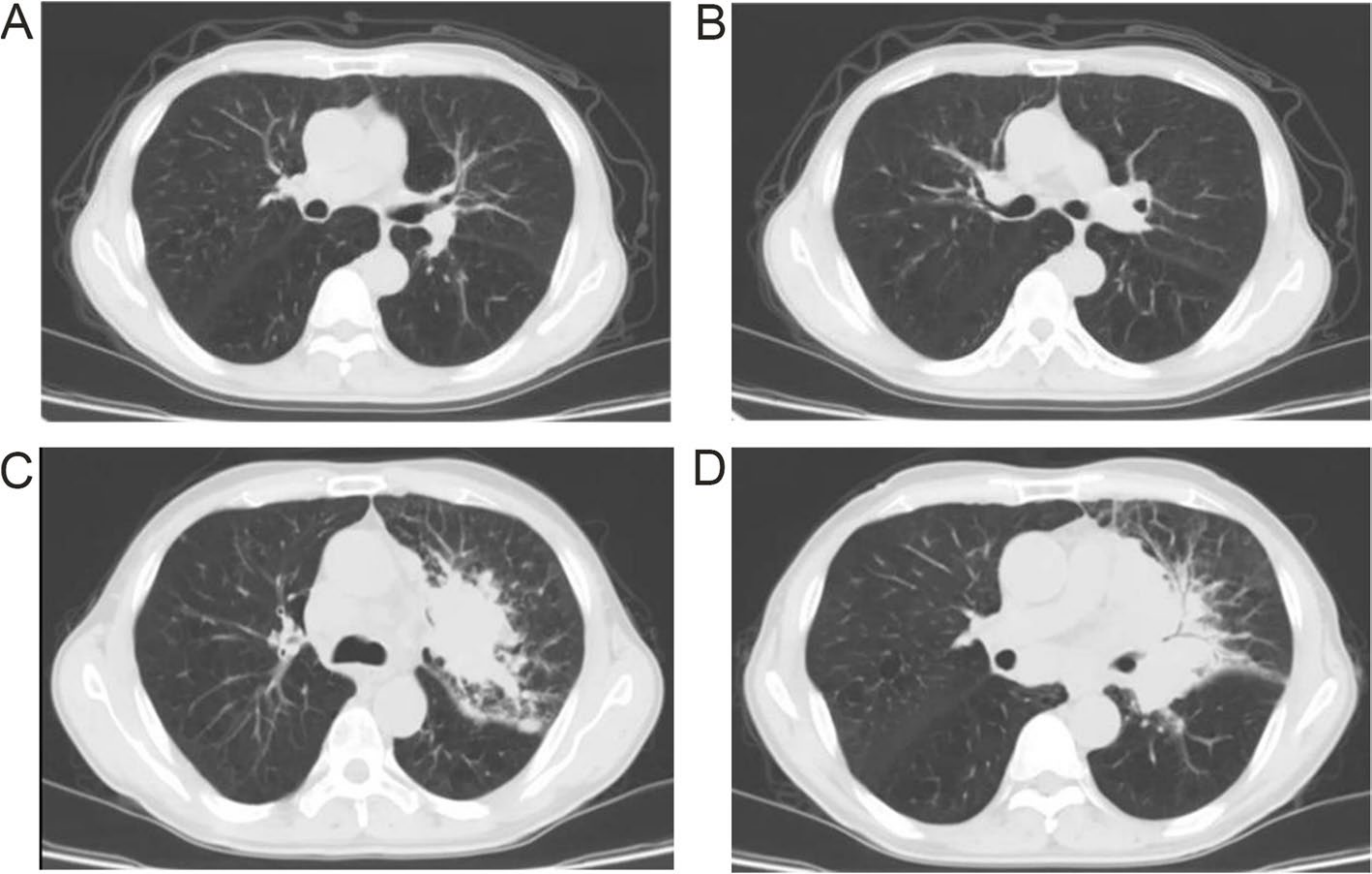
**A** and **B** show the CT scan results 2 months after treatment. **C** and **D** show the CT scan results after disease progressed

The patient intravenously received cisplatin (75 mg/m^2^) and etoposide (80 mg/m^2^ on days 1–3) and durvalumab (humanized monoclonal PD-L1 inhibitor, 1500 mg) every 3 weeks for four cycles. 2 months after treatment, the patient achieved partial remission (PR) in imaging assessment (Fig. 3A and B), and the patient refused prophylactic cranial irradiation and chest radiotherapy. Durvalumab (1500 mg) was administered once every four weeks, and the disease progressed after four times of maintenance treatment (Fig. 3C and D). The patient refused chemotherapy again, immunotherapy and radiotherapy, anlotinib and analgesic drugs orally only, and went home to hospice care. He succumbed to his disease approximately 9 months after his diagnosis.

## Discussion

The patient was hospitalized with CAP for the first time in June, which was not the frequent season of CAP [3], and the blood routine indicated that the lymph number was significantly decreased, indicating weakened immunity. Although the initial diagnosis was CAP and antibacterial therapy was given, unfortunately, no pathogenic bacteria were detected through sputum culture and alveolar lavage fluid test, which may be related to the use of antibacterial drugs immediately after admission. Complete absorption of the right upper lung lesion on CT in the second admission also confirmed the diagnosis of CAP. Twelve previous case–control studies from Europe and Canada found that a positive relationship was observed between lung cancer and pneumonia diagnosed 2 years or less before lung cancer for men [4]. Due to good condition after discharge, follow-up and CT recheck were not conducted as required, and the community doctors did not pay much attention to the correlation between pneumonia and lung cancer, not to mention that he had a history of heavy smoking and COPD. About 7 months after discharge, the patient returned to our hospital with an incredible mass on chest CT. Suggesting that unprovoked pneumonia occurs in COPD is likely a manifestation of weakened immunity and may be a precursor to tumor development. To our knowledge, this is the first case we've seen in the real world in a patient with COPD that went from cancer-free to ES-SCLC in less than 7 months. After diagnosis of ES-SCLC, EP and durvalumab were selected as the first-line regimen. The patient was generally in good condition at the first diagnosis of ES-SCLC. Given the rapid growth of the lesions, combined radiotherapy was recommended, but the patient refused. After 2 months of treatment, although the lesions were significantly reduced, late maintenance therapy with durvalumab alone could not stop the crazy progression of cancer. Due to severe disappointment, depression, and debility progression, the patient was only willing to receive anlotinib treatment and died about 2 months later. If anlotinib combined with chemotherapy or standardized regimens of different immune checkpoint inhibitors, as well as combined with radiotherapy and/or cellular therapy may have longer overall survival after progression [5–7].

Lung cancer and COPD share common pathogenic factors. Lung cancer is the main cause of death in COPD, and COPD is also a risk factor for lung cancer [1, 8]. However, the underlying associations and molecular pathological mechanisms between COPD and SCLC remain unclear, and finding effective ways to prevent, diagnose, and treat SCLC in patients with COPD is an urgent task. The patient's greatest hazard was a history of heavy smoking, as there were no other cancer risk factors other than COPD and age.Compared with non-COPD subjects, smoking behaviors showed a significantly higher effect on SCLC risk among COPD subjects, and further, COPD patients showed a 1.86-fold higher risk of SCLC [9]. The relationship between COPD and its different phenotypes and lung cancer has also been reported: the degree of COPD severity, including airflow obstruction, visual emphysema, and respiratory exacerbations, was independently predictive of lung cancer [10]. Some scholars revealed that it seems unlikely that COPD is an independent risk factor for lung cancer [11]. SCLC has a high degree of malignancy, rapid growth, easy recurrence and short survival [7], which can be interpreted incisively and vividly in this patient. Previous studies with small samples have also found that tumors grow rapidly in patients with COPD and idiopathic interstitial pneumonia, and SCLC is significantly correlated with rapid tumor growth [12]. However, up to now, we have not found any reports on tumor volume doubling time (VDT) in COPD combined with SCLC. There is also a lack of research on the mechanism of COPD combined with SCLC. In the future, in addition to revealing the potential biological mechanism of comorbidities of COPD and SCLC, large prospective cohort studies are also needed to further reveal and verify the association between COPD and SCLC's rapid growth. It is also expected that with the help of big data and artificial intelligence, the VDT of different diseases combined with different types of lung cancer can be measured and the risk factors of lung cancer can be included to establish an accurate prediction model, so as to provide scientific and reliable CT follow-up interval for such patients and prevent the recurrence of such tragic events.

## Conclusions

For COPD patients with immunocompromised manifestations, it is necessary to be alert to complications and shorten the follow-up interval of chest CT. COPD may accelerate the formation and progression of SCLC.

## Data Availability

The original contributions presented in the study are included in the article. Further inquiries can be directed to the corresponding author.

## Abbreviation

COPD: Chronic obstructive pulmonary disease
NSE: Neuron-specific enolase
ES-SCLC: Extensive small cell lung cancer
